# Application of the Briggs–Rauscher Oscillatory Reaction for Tartrazine Determination in Food Dye: Spectroscopic, Microscopic, and Analytical Characterization

**DOI:** 10.3390/foods15071181

**Published:** 2026-04-01

**Authors:** Jelena V. Senćanski, Jelena P. Maksimović, Danica V. Bajuk-Bogdanović, Aleksandra M. Radulović, Tihomir V. Jevtić, Nebojša I. Potkonjak, Maja C. Pagnacco

**Affiliations:** 1 Institute for Multidisciplinary Research, National Institute of the Republic of Serbia, University of Belgrade, Kneza Višeslava 1, 11030 Belgrade, Serbia; jelena.sencanski@imsi.bg.ac.rs; 2 Faculty of Physical Chemistry, University of Belgrade, Studentski Trg 12–15, 11000 Belgrade, Serbia; jelena.maksimovic@ffh.bg.ac.rs (J.P.M.); danabb@ffh.bg.ac.rs (D.V.B.-B.); tihomirposao@hotmail.com (T.V.J.); 3 Institute of General and Physical Chemistry, University of Belgrade, Studentski Trg 12–15, 11000 Belgrade, Serbia; aradulovic@iofh.bg.ac.rs; 4 VINČA Institute of Nuclear Sciences—National Institute of the Republic of Serbia, University of Belgrade, Mike Petrovica Alasa 12–14, 11000 Belgrade, Serbia; 5 Institute of Chemistry, Technology and Metallurgy, National Institute of the Republic of Serbia, University of Belgrade, Njegoševa 12, 11000 Belgrade, Serbia

**Keywords:** food dye, azo dye, tartrazine, microconcentration, oscillatory reaction, Briggs-Rauscher reaction, analytical methods

## Abstract

Tartrazine (E102), a synthetic azo dye, is extensively utilized across diverse industrial sectors. Understanding the mechanisms of tartrazine degradation and identifying its breakdown products are essential for assessing its environmental fate and potential health risks. Tartrazine is studied in this work in terms of: (i) determining its concentration in a commercial food dye by use the Briggs–Rauscher (BR) oscillatory (clock) reaction as seldom-employed analytical method, (ii) examining its degradation in a highly oxidative system, such as the BR oscillatory reaction, using Raman and FTIR spectroscopy, and (iii) monitoring the degradation process in the BR system at different magnifications using optical and scanning electron microscopy (SEM). The limits of detection (LOD) and quantification (LOQ) obtained for the BR reaction were higher than those determined by UV-Vis spectroscopy. Both methods determined comparable concentrations of tartrazine in the food dye. Based on the results obtained, the reaction mechanism for tartrazine degradation in the clock reaction was proposed. The findings strongly support the BR reaction as an easily available method for determining unknown concentrations of tartrazine in commercial food dyes. Furthermore, this study highlights the potential of the BR reaction for determining microconcentrations and for the rapid degradation of commercial food dyes.

## 1. Introduction

Synthetic azo dyes are widely used as additives in the food, cosmetic, textile, and pharmaceutical industries due to their bright coloration, stability, and low production costs [1,2,3,4,5,6,7]. Among them, tartrazine (E102) is one of the most common yellow azo dyes, characterized by sulfonic acid groups that are highly polar and confer high water solubility [2,3,4,5,6,7].

The studies regarding tartrazine’s toxic effect on living organisms (e.g., rats) showed that it could be highly poisonous, and it can be a cancer trigger [8]. The corresponding acceptable daily intake (ADI) is 7.5 mg/kg body weight (bw)/day [9]. This fact is extremely important when the accumulation of additives in living organisms is considered.

The Briggs–Rauscher (BR) reaction is well known as a high oxidation medium [10,11]. This clock reaction is abundant in radical species such as HO_●_, HOO_●_, and I_●_ IO_2__●_ [12,13]. Furthermore, due to the BR reaction occurring at pH ≈ 2 (the acidity of the stomach) and at 37 °C (physiological temperature), it is a suitable model system to evaluate the mechanisms underlying tartrazine degradation in the digestive system. Understanding the mechanisms of tartrazine degradation and identifying its breakdown products are essential for evaluating its potential impact on humans. The BR reaction is bypassed in favor of traditional photocatalytic [14] and Fenton-like [15] reactions, thereby failing to reach its full potential. Recently, the BR reaction was successfully applied for determining indigo carmine in food dye [16]. The same study reports better BR reaction analytical validation parameters (sensitivity, LOD, and LOQ) regarding the UV-Vis method.

This study introduces a combined analytical and degradation approach based on the BR oscillatory reaction for the investigation of tartrazine. While the BR reaction has previously been applied to selected colorants, its application to tartrazine has not been reported. In this work, the BR system is used in a dual role: (i) as an unconventional analytical tool for estimating tartrazine concentration in a commercial food dye, and (ii) as a highly oxidative medium for probing its degradation behavior. In addition, a multi-technique characterization strategy (UV-Vis, Raman, FTIR, optical microscopy, and SEM) is employed to provide complementary insight into both analytical response and structural changes within the BR system. Particular emphasis is placed on FTIR-based identification of functional group transformations, contributing to a tentative description of degradation pathways under oscillatory conditions.

## 2. Materials and Methods

### 2.1. The Experimental Conditions for the Clock Briggs–Rauscher Reaction

All experiments were performed in a closed, thermostated (T = 37.1 °C), well-stirred reactor (σ = 900 rpm). The time evolution of the BR system was monitored potentiometrically with an ion-sensitive I^−^ electrode as the working electrode and a Hg/HgSO_4_ electrode as the reference.

The initial concentrations of the reactants were: [Malonic acid]_0_ = 0.0789 mol dm^−3^, [MnSO_4_]_0_ = 0.00752 mol dm^−3^, [HClO_4_]_0_ = 0.03 mol dm^−3^, [KIO_3_]_0_ = 0.0752 mol dm^−3^, [H_2_O_2_]_0_ = 1.176 mol dm^−3^. 50 µL of Tartrazine solutions of different concentrations (5.86 × 10^−4^ mol dm^−3^; 2.84 × 10^−2^ mol dm^−3^; 5.71 × 10^−2^ mol dm^−3^; 8.52 × 10^−2^ mol dm^−3^; 1.13 × 10^−1^ mol dm^−3^; 1.69 × 10^−1^ mol dm^−3^; 2.25 × 10^−1^ mol dm^−3^; 2.80 × 10^−4^ mol dm^−3^) were added to the reaction mixture 30 s after the initiation of the reaction with hydrogen peroxide.

The commercial food dye (mixture of tartrazine and dextrose), 3 g, was dissolved in 10 mL of distilled water at room temperature. After that, 50 µL of the solution obtained was added to the Briggs–Rauscher reaction after 30 s.

All experiments were performed at least in triplicate under identical conditions, and the reported values represent mean values. The reproducibility of the oscillatory period (τ_osc_) was evaluated, and variations between repeated measurements were found to be low, confirming the stability of the experimental setup.

### 2.2. UV-Vis Measurements

#### Standard Calibration Curve of Tartrazine

The UV/Vis calibration curve was obtained by recording absorbance at decreasing concentrations of the main solution, prepared by dissolving 0.0157 g of standard tartrazine (Sigma-Aldrich, St. Louis, MO, USA) in 100 mL of distilled water. The main solution was diluted 5, 10, 25, 50, and 100× times to obtain an analytical curve. The sample (commercially available food dye in the Serbian market) was made by dissolving 0.0445 g in 100 mL of distilled water. An Agilent 8453 spectrophotometer (Agilent Technologies, Inc. Santa Clara, CA, USA) was used over 200–1100 nm at ambient temperature to obtain the analytical curve at circa 25 °C. Calibration curves were constructed using multiple concentration levels, and each point represents the mean value of repeated measurements.

### 2.3. The Preparation of the Samples for Micro-Raman and FTIR-ATR Spectrometry, Optical, and SEM Microscopy

To examine the BR reaction with tartrazine by Micro Raman, FTIR, optical, and SEM microscopes, each component of the BR reaction was evaporated in separate Petri dishes. Also, the mixture of all BR reaction components (0.7 mL [Malonic acid] = 0.282 mol dm^−3^; 0.5 mL [MnSO_4_] = 0.0376 mol dm^−3^; 0.5 mL [HClO_4_] = 0.15 mol dm^−3^; 0.5 mL [KIO_3_] = 0.376 mol dm^−3^ and 0.3 mL [H_2_O_2_] = 9.8 mol dm^−3^) and the mixture of all BR reaction components with tartrazine (0.001 g) were also undergone evaporation in separate Petri dishes. Finally, tartrazine itself (0.001 g in 1 mL of distilled water) was also obtained by evaporation. The evaporation was carried out at approximately 25 °C for 2 days under atmospheric pressure.

### 2.4. Micro-Raman Spectrometry

To record Raman spectra of the BR oscillatory reaction, its single components, and tartrazine in the BR reaction, the samples were dried onto Petri dishes, and the residues were analyzed by Raman spectroscopy. The spectra of all samples (components of the BR reaction, the BR reaction itself, standard tartrazine (Sigma Aldrich), and tartrazine in the BR reaction) were recorded by a Thermo Scientific DXR Raman Microscope (Thermo Scientific Inc., Waltham, MA, USA). The commercially available food dye containing tartrazine (commercially available food dye, Belgrade) was also recorded. The spectra were recorded at λ = 532 nm, laser power 1 mW, magnification 10×, and a 2.1 µm laser spot size.

### 2.5. FTIR Spectroscopy-ATR

Perkin Elmer equipment, ATR, was used to record spectra of samples evaporated (components of the BR reaction, the BR reaction itself, standard tartrazine (Sigma Aldrich), and tartrazine in the BR reaction) in the range of 4000–400 cm^−1^ at 20 scans and a spectral resolution of 2 cm^−1^.

### 2.6. Optical Microscope

The Geefantech transmission microscope was used to carry out the analysis of all samples evaporated (components of the BR reaction, the BR reaction itself, standard tartrazine (Sigma Aldrich), and tartrazine in the BR reaction) under the magnifications ranging from 160× to 400 × to 1600×.

### 2.7. Scanning Electron Microscopy (SEM)

Images were captured using a JEOL JSM 6460 LV microscope (JEOL Ltd., Tokyo, Japan). For preparation, samples were mounted onto specimen stubs with adhesive tape and subsequently gold-coated using a BAL-TEC SCD 005 sputter coater (Bal-Tec SCD, Balzers, Liechtenstein) to ensure electrical conductivity and prevent charging artifacts. The particles’ sizes were determined my SemAfore 5.21 software. The particular samples (single components of the BR reaction, the BR reaction, tartrazine, and tartrazine in the BR reaction) are evaporated at circa 25 °C for 2 days in Petri dishes and subjected to optical microscopy. Several regions of each sample were examined in order to ensure that the observed morphology is representative. The presented micrographs correspond to the most typical structures observed across different areas of the samples. The selected magnifications were chosen to capture general morphological features of the systems, rather than fine nanoscale details.

## 3. Results

### 3.1. The Tartrazine Influence on Briggs–Rauscher Oscillatory Reaction Dynamics

**Figure 1:**
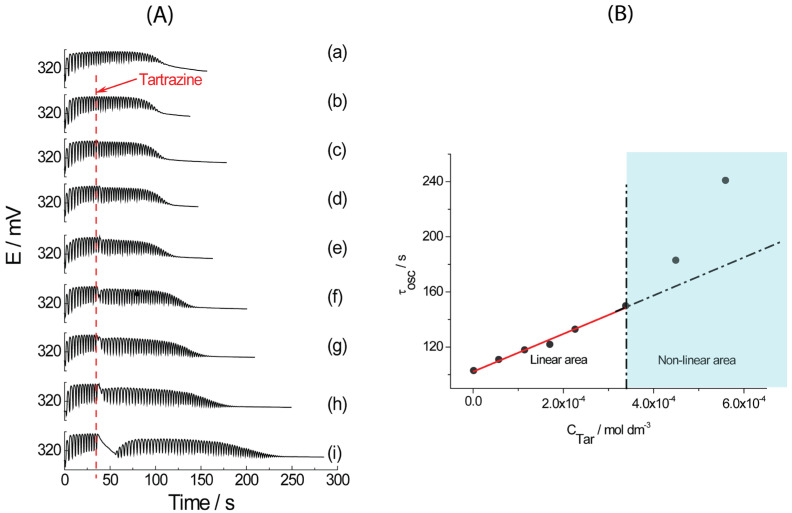
(**A**) Basic BR oscillogram (a) and BR oscillograms obtained with the addition of various concentrations of tartrazine: 1.17 × 10^−6^ mol dm^−3^ (b), 5.67 × 10^−5^ mol dm^−3^ (c), 1.14 × 10^−4^ mol dm^−3^ (d), 1.70 × 10^−4^ mol dm^−3^ (e), 2.26 × 10^−4^ mol dm^−3^ (f), 3.38 × 10^−4^ mol dm^−3^ (g), 4.49 × 10^−4^ mol dm^−3^ (h), and 5.59 × 10^−4^ mol dm^−3^ (i). (**B**) The BR oscillatory period, τ_osc_, dependence on the tartrazine concentration.

The challenge of this study was to investigate the influence of tartrazine addition on the dynamics of the BR reaction. It was done by dissolving both samples in water. Injections of standard and dissolved commercial food dye into the BR system were performed as described in Section 2. The influence of tartrazine on BR dynamics, specifically the oscillatory period τ_osc_, is further investigated and discussed. The tartrazine effects on BR oscillatory dynamics, compared to a basic one (BR oscillogram not containing tartrazine), are presented in Figure 1A. The addition of tartrazine to the BR reaction increased the oscillatory period, τ_osc_ (the duration of the whole oscillogram). Furthermore, the addition of tartrazine standard aqueous solution (higher than 3.38 × 10^−4^ mol dm^−3^) to the oscillatory BR reaction caused quenching of oscillatory dynamics (temporarily stopping the oscillations). When the BR oscillatory period, τ_osc_ was presented as a function of tartrazine concentration (1.17 × 10^−6^ mol dm^−3^, 5.67 × 10^−5^ mol dm^−3^, 1.14 × 10^−4^ mol dm^−3^, 1.70 × 10^−4^ mol dm^−3^, 2.26 × 10^−4^ mol dm^−3^, 3.38 × 10^−4^ mol dm^−3^, 4.49 × 10^−4^ mol dm^−3^, 5.59 × 10^−4^ mol dm^−3^) in the BR system, the two regions were obtained (see Figure 1B). The first one can be used as an analytical curve, τ_osc_ = (137,000 ± 8000) (C + (102 ± 2)), and the second one shows non-linear behavior, as noted in Figure 1B. The concentration, C, is given in mol dm^−3^.

The study aimed to determine the unknown concentration of tartrazine in a commercial food dye (a mixture of tartrazine and dextrose) using the BR reaction. Based on Pagnacco et al. [16], dextrose does not affect the Briggs–Rauscher dynamics, so the signal of quenching the BR reaction was attributed only to tartrazine. Therefore, the BR oscillatory period obtained when the commercial food dye was dissolved in water (water does not influence the oscillatory reaction in a small volume, e.g., 50 µL) and injected into the BR system, could be connected only with the tartrazine concentration. The measured BR oscillatory period was interpolated into the analytical curve. After calculation, the tartrazine content of 3.3% was obtained for the commercial food dye sample. The result obtained by the BR reaction was compared to the one obtained by the standard UV-Vis technique (Section 3.2).

### 3.2. UV-Vis Spectrometry-Determining Tartrazine Concentration in a Commercial Food Dye

**Figure 2:**
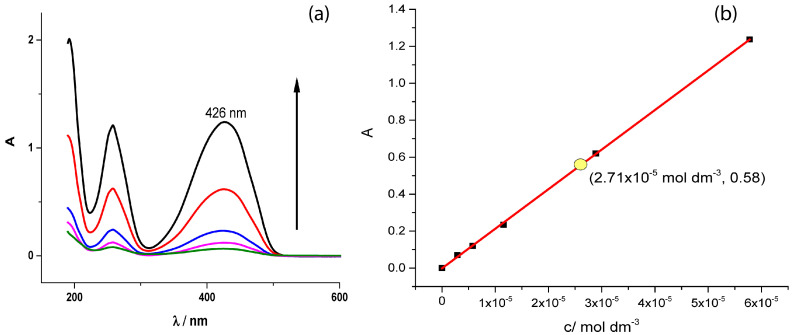
(**a**) The standard tartrazine water solutions UV Vis spectra 5.78 × 10^−5^ mol dm^−3^, 2.89 × 10^−5^ mol dm^−3^, 1.16 × 10^−5^ mol dm^−3^, 5.78 × 10^−6^ mol dm^−3,^ and 2.89 × 10^−6^ mol dm^−3^ (**b**), and analytical UV VIS curve at 426 nm.

The UV-Vis spectra of standard tartrazine water solutions are presented in Figure 2a. Two wide peaks are observed at 258 nm and 426 nm. The peak at 426 nm is used to obtain the tartrazine analytical curve A_426_ = (21,400 ± 200) × C + (0.001 ± 0.005), where C is in mol dm^−3^. The molar absorption coefficient obtained here, 2.14 (104 mol dm^−3^ cm^−1^, meets a similar result reported in [17], 2.16 (104 mol dm^−3^ cm^−1^ in water solution, indicating reproducibility. The commercially available food dye analyzed in this work is a mixture of tartrazine and dextrose. Dextrose does not exhibit a UV-Vis spectrum because it lacks chromophores. As a consequence, tartrazine in commercially available food dye water solution could be directly analyzed by the UV-Vis method. Therefore, the standard analytical curve was used to determine the unknown tartrazine concentration in a commercial food dye by interpolating the measured absorbance (Figure 2b; the yellow point noted in the analytical curve with the presented coordinates).

The tartrazine concentration determined in the commercial food dye dissolved was C = 2.71 × 10^−5^ mol dm^−3^ for A = 0.58 ± 0.02, containing 3.25% of tartrazine in the commercial food dye. Taking into account that an equivalent result was obtained in the Briggs–Rauscher analysis, this makes the BR reaction a promising method to determine unknown concentrations of tartrazine in such samples.

### 3.3. The Comparative Analysis Between the BR and UV-Vis Methods for Determining Tartrazine

The comparison of the BR reaction, a seldom-used method for determining colorant concentrations, with UV-Vis, a standard method, is based on analytical validation parameters (sensitivity, LOD, and LOQ). If sensitivity is defined as the slope of an analytical curve [18], the BR reaction shows higher sensitivity due to its steeper slope compared to the UV-Vis method (Table 1). It means that the BR reaction has a better ability to distinguish small differences in tartrazine concentrations. The limit of detection (LOD) value is the lowest concentration of the analyte detectable at a specified level of confidence. The limit of quantification (LOQ) is the limit of the lowest concentration determinable but maintains an acceptable level of repeatable precision and trueness [13]. The Briggs–Rauscher and UV-Vis LODs and LOQs were calculated according to the Formulae (1) and (2),

(1) $$LOD=3.3\times \frac{\delta }{slope}$$

(2) $$LOQ=10\times \frac{\delta }{slope}$$

where *δ* is defined by Formula (3),

(3) $$\delta =\sqrt{\frac{{\Sigma \left(yi-y_{exp}\right)}^{2}}{\left(n-1\right)}}$$

where *n* is the number of measurements and *y* is the oscillatory period for the BR method, while for the UV-Vis method, it is absorbance. These parameters are listed in Table 1.

Better LOD and LOQ are obtained for the UV-Vis method. The LOQ is about a hundred times lower for UV-Vis than for the BR method. This is a consequence of lower concentrations included than for the BR reaction to obtain an analytical curve by this method, as well as a lower δ obtained, due to better repeatability and trueness.

The calculated analytical parameters are based on repeated measurements, ensuring reliable estimation of sensitivity, LOD, and LOQ values.

However, the comparable tartrazine percentage values obtained by both analytical methods classify the BR reaction as a promising method for determining colorants in foods.

### 3.4. Raman Spectroscopy Characterization of the BR Reaction, Its Components, and Tartrazine Added in the BR Reaction

**Figure 3:**
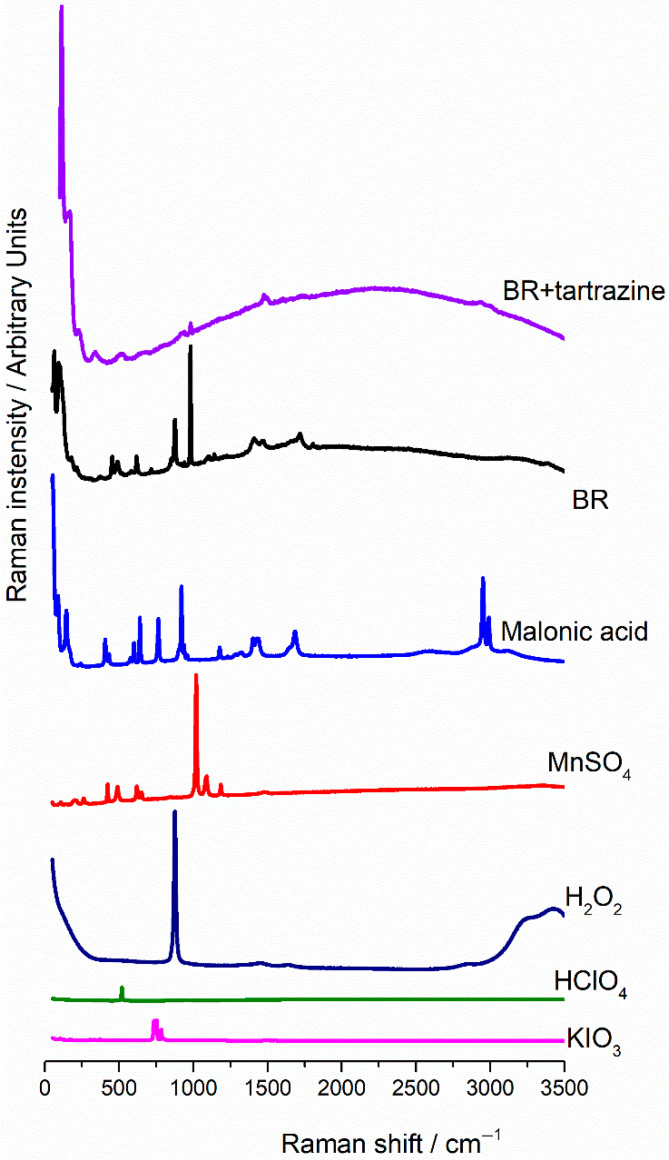
Raman spectra of the BR reaction, its components, and tartrazine added in the BR reaction (evaporated samples).

The Raman spectra of each Briggs–Rauscher reaction component, as well as the BR reaction itself, and the BR reaction with the addition of tartrazine, all evaporated at room temperature for two days, are presented in Figure 3.

Chowdhry et al. were the first to attempt to record Raman spectra of the BR reaction over time [19]. Only the intensive peak at 160 cm^−1^ was monitored in time and identified as a band of polyiodide chains in the starch–iodine complex. Given that starch is not present in the BR system investigated in this study, this peak is not observed in the Raman spectrum. The Raman spectrum of the BR oscillatory system recorded in this study shows peaks at 66, 99, 170, 220, 367, 455, 488, 613, 718, 870, 981, 1410, 1467, and 1719 cm^−1^, corresponding to malonic acid, MnSO_4,_ and KIO_3_ present in high excess.

However, a wide peak (from 500 to 3500 cm^−1^) is observed in both studies (Chowdhry et al. [19] and this one), which is due to fluorescence in the BR system (Figure 3, black line). Furthermore, the fluorescence increased when tartrazine was added to the BR system, resulting in a wider peak, as shown in Figure 3 (violet line). It supports the conclusion that tartrazine itself could not be detected because of its strong fluorescence under the same equipment conditions. Due to the strong, pristine fluorescence of tartrazine, the baseline is shifted upward and shows a broad maximum in the spectrum BR with tartrazine added (Figure 3, violet). After tartrazine addition, most of the peaks observed in the BR oscillatory reaction disappeared, probably due to high fluorescence obscuring their positions, leaving only peaks at lower wavenumbers (<450 cm^−1^) belonging to malonic acid (or iodomalonic acid).

Given that Raman-inactive vibrations may be visible in the FTIR spectrum, the same spectra were recorded for each sample.

### 3.5. FTIR Spectroscopy Characterization of the BR Reaction, Its Components, and Tartrazine Added in the BR Reaction

**Figure 4:**
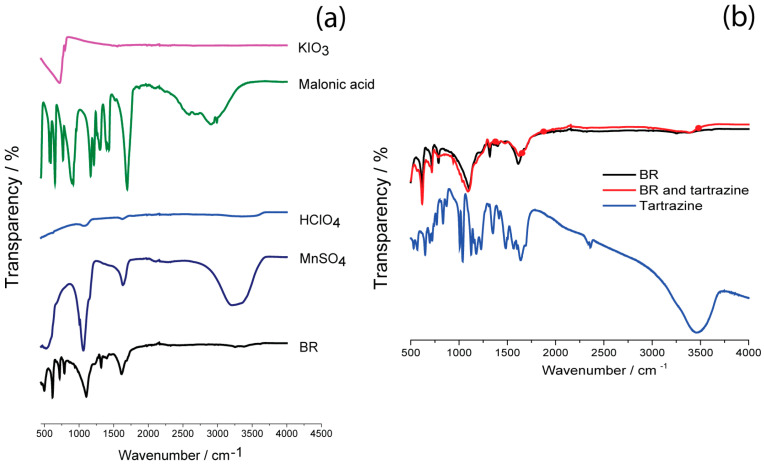
FTIR spectra of evaporated (**a**) single components of the BR reaction (KIO_3_, Malonic acid, HClO_4_, MnSO_4_) and the BR reaction itself; (**b**) tartrazine, tartrazine in the BR reaction and the BR reaction.

The evaporated samples of the whole BR reaction, each BR component, tartrazine, and the BR reaction with the addition of tartrazine (BR plus tartrazine) were also characterized by FTIR in this study. The evaporation was performed at room temperature for 2 days to prepare these samples for FTIR analysis, aiming to detect the products of the tartrazine reaction with the BR mixture. This study also provides, for the first time, an investigation of the entire BR reaction system by FTIR (Figure 4). According to the literature, precipitates obtained in the BR reaction and other analytes added to the BR system were usually analyzed by FTIR [20,21].

FTIR spectra presented in Figure 4a list the peaks belonging to BR reaction components: HClO_4_ (the peaks positioned at 1112 and 1617 cm^−1^); 721 cm^−1^ originating from KIO_3_; 1629, 1344, 1101, 788, and 514 cm^−1^, which malonic acid possesses; and 445, 1066, 1629, and 3190 cm^−1^ visible in MnSO_4_ catalyst’s spectrum. After the addition of tartrazine to the BR system, the spectrum of the BR reaction (black line) changed to the spectrum shown in Figure 4b (red line). The baseline of the BR system with tartrazine is almost unchanged with regard to the FTIR spectrum of the BR system, which means that tartrazine decomposes in the BR reaction. After the addition of tartrazine (red spectrum in Figure 4b), the lines observable at 721 cm^−1^ belonging to KIO_3_ cm^−1^ decreased, as well as the lines positioned at 541 cm^−1^ and 788 cm^−1,^ which belong to malonic acid. The lines of tartrazine at 1639, 1596, 1565, 1500, 1484 cm^−1^ (blue spectrum in Figure 4b) are not present in the red spectrum (BR with tartrazine), meaning that the –N=N– bond from tartrazine has been broken up [22]. The presence of >S=O at 1035 cm^−1^, and C_6_H_5_–, C_6_H_5_–N=N–, C_6_H_5_–SO_2_– at 1482 cm^−1^ [22], is evident in the red spectrum (BR with tartrazine). The peak positioned at 1689 cm^−1^ means that some carboxylic acids [23] are present in the red spectrum (BR with tartrazine).

### 3.6. The Proposed Mechanism of Tartrazine Degradation in the Briggs–Rauscher Reaction

**Figure 5:**
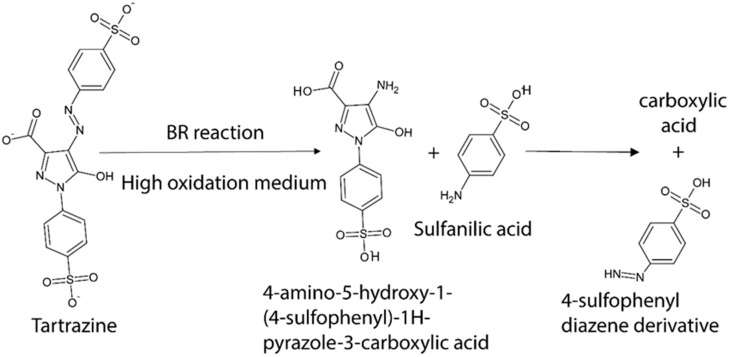
Proposed reaction mechanism of tartrazine degradation in the BR system based on FTIR observations.

Based on FTIR analysis, a tentative degradation pathway of tartrazine in the BR reaction is proposed, as shown in Figure 5. The FTIR results (Section 3.5) indicate significant changes in functional groups during the reaction. In particular, the disappearance of the characteristic –N=N– band (around 1639 cm^−1^) suggests cleavage of the azo bond, accompanied by the appearance of bands corresponding to >S=O and aromatic structures.

These observations suggest that reactive species present in the BR system (e.g., HO_●_ and HOO_●_ radicals) may attack the azo linkage, leading to fragmentation of the tartrazine molecule. The formation of aromatic sulfonic and amino-containing derivatives may occur, in agreement with previously reported degradation pathways under oxidative conditions. Further transformation of these intermediates may result in smaller oxygenated compounds, such as carboxylic acids, and ultimately mineralization products (CO_2_ and H_2_O).

Tartrazine is known to undergo both oxidative and reductive degradation pathways. Previous studies have reported the formation of aromatic sulfonic acids, amino derivatives, and low-molecular-weight organic acids under various advanced oxidation processes [24,25,26,27,28]. The trends observed in this study are consistent with these literature reports, supporting the proposed transformation scheme.

It should be noted that the proposed degradation pathway is based primarily on FTIR spectral changes and therefore reflects functional group transformations rather than definitive identification of individual intermediates. While FTIR provides evidence for azo bond cleavage and the formation of oxygen-containing groups, it does not allow unambiguous assignment of specific molecular structures. Therefore, the suggested intermediates should be considered tentative and supported by literature comparisons. A more detailed elucidation of reaction intermediates would require complementary techniques such as LC–MS or GC–MS, which are beyond the scope of the present study.

### 3.7. The Microscopic Characterization of the BR Reaction and Its Components, Tartrazine, and the BR Reaction Containing Tartrazine

**Figure 6:**
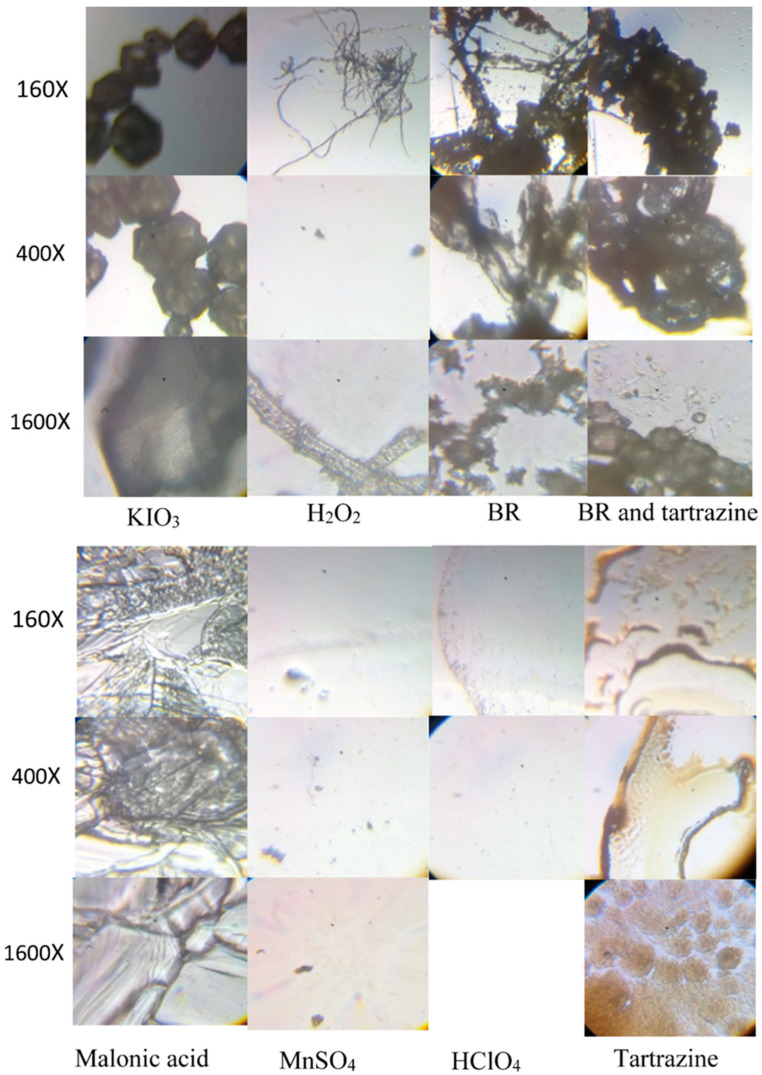
The pure components of the BR reaction, the tartrazine in the BR reaction, and tartrazine under the 160×, 400×, and 1600× magnifications.

To have better insight into the BR reaction and its single components, tartrazine, and the BR reaction containing tartrazine, the evaporated samples were subjected to microscopic analysis, under magnifications ranging from 160 to 400 to 1600×. The obtained pictures are shown in Figure 6.

Optical microscopic characterization shows hexagonal particles of potassium iodate, a chain morphology of malonic acid, and a spider web appearance of H_2_O_2_ (see Figure 6). BR reaction consists of agglomerated particles containing partly defined hexagonal granules and a chain morphology with snowflake features (Figure 6). Tartrazine itself possesses granules full of small needles (Figure 6), while the mixture of tartrazine and BR reaction shows hexagonal granules that become more agglomerated compared to the BR ones (Figure 6).

**Figure 7:**
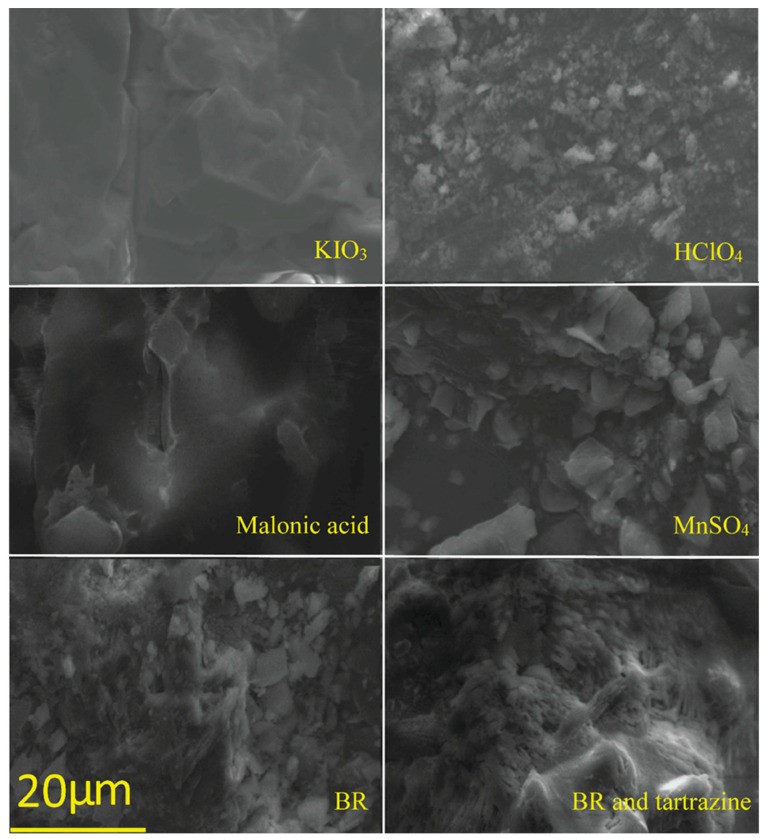
SEM micrographs of pure components evaporated samples forming the BR reaction, BR reaction evaporated sample, and tartrazine in the BR reaction evaporated sample.

Given that optical microscopy can observe only the surface of the system, to gain better insight into the system morphology, SEM analysis of the evaporated samples was carried out, as shown in Figure 7. The evaporated samples of the pure components of the BR reaction, and the tartrazine in the BR reaction, were subjected to SEM analysis.

SEM pictures revealed that KIO_3_ has a fractured plate morphology with well-defined plate edges, ranging from 4 to 12 µm, while HClO_4_ has a graveled morphology, ranging from 0.7 to 4 µm. Malonic acid shows large, broken particles, while MnSO_4_ has irregular particles 2–7 µm, and BR has a gravelly morphology with particles ranging from 0.9 to 6 µm. The BR morphology is similar to the mixture morphologies of HClO_4_ and MnSO_4_. When tartrazine was added to the BR system, the BR morphology changed to a microporous form, with 0.3 to 1.3 micrometer-sized particles and 1.9–1.4 micrometer pores. The change in morphology indicates that some reaction between components of the BR reaction and tartrazine had occurred (Figure 7). The observed morphologies were consistent across multiple analyzed regions.

## 4. Conclusions

This study demonstrates the applicability of the BR oscillatory reaction for the determination and transformation of tartrazine in a commercial food dye. The tartrazine content obtained using the BR method (3.3%) was in good agreement with the value determined by UV-Vis spectroscopy (3.25%), indicating that the oscillatory system can provide reliable concentration estimates under the applied conditions. Despite its lower sensitivity compared to UV-Vis (higher LOD and LOQ values), the BR reaction offers a simple and unconventional alternative approach based on changes in oscillatory dynamics, which may be useful for rapid screening or complementary analysis. Spectroscopic results suggest that tartrazine undergoes oxidative degradation in the BR system, primarily through azo bond cleavage and subsequent formation of oxygen-containing functional groups. The proposed pathway, supported by FTIR analysis and literature data, provides insight at the level of functional group transformations rather than definitive identification of individual intermediates. Microscopic observations indicate morphological changes in the BR system upon tartrazine addition, reflecting structural reorganization of the reaction products, although these findings should be interpreted qualitatively. Overall, the results highlight the dual role of the BR reaction as both an analytical and reactive system. Further studies employing complementary techniques such as mass spectrometry would be required to confirm the identity of degradation products and to fully elucidate the reaction mechanism.

## Figures and Tables

**Table 1:** The comparison between two methods to determine tartrazine in the food dye.

Curve Equation	BR Reaction τ_osc_ = (137,000 ± 8000) × C + (102 ± 2)	UV-Vis Method A = (21,400 ± 200) × C + (0.001 ± 0.005)
LOD	(4.3 ± 0.3) × 10^−5^	(1.1 ± 0.1) × 10^−6^
LOQ	(1.3 ± 0.1) × 10^−4^	(3.0 ± 0.2) × 10^−6^

## Data Availability

The original contributions presented in this study are included in the article. Further inquiries can be directed to the corresponding authors.
